# Behavioral activation / inhibition systems and lifestyle as predictors of mental disorders in adolescent athletes during Covid19 pandemic

**DOI:** 10.1186/s12889-022-13816-3

**Published:** 2022-07-29

**Authors:** Morteza Homayounnia Firoozjah, Alireza Homayouni, Shahnaz Shahrbanian, Shaghayegh Shahriari, Diana Janinejad

**Affiliations:** 1 Department of Physical Education, Farhangian University, Tehran, Iran; 2 Department of Psychology, Bandargaz Branch, Islamic Azad University, Bandargaz, Iran; 3 Department of Sport Science, Faculty of Humanities, Tarbiat Modarres University, Tehran, Iran; 4 Undergraduate Psychology/Cognitive Sciences Researcher, University of California Irvine, California, USA

**Keywords:** Behavioral systems, Lifestyle, Mental disorders, Adolescent athletes, Covid19 pandemic

## Abstract

The following study investigates the correlational relationship between behavioral activation/inhibition systems, lifestyle and mental disorders in Adolescent Athletes during the Covid-19 pandemic. Methods: Research methods are descriptive and correlational; “Of the eligible participants who were available during a COVID-19 quarantine period from June through August 2020 (*N* = 180), the Krejcie and Morgan Sampling Method was used to simplify the process of determining the sample size for a finite population [46], resulting in a calculation of N = 130 sample participants. to respond to Carver & White’s Behavioral activation/inhibition systems Scale (BIS/BAS), Mille’s Lifestyle Questionnaire and Goldberg & Williams’s General Health Questionnaire (GHQ-12). Data was analyzed using linear regression analysis and Pearson’s correlation coefficient. Results: Findings showed a positive correlation of statistical significance between behavioral inhibition systems (BIS) and mental disorders in Adolescent Athletes at the 0.01 level and a negative correlation of statistical significance between scaling components of the behavioral activation systems (BAS), lifestyle and mental disorders in Adolescent Athletes at the 0.05 level. Conclusions: Analyzing the data, it can thus be concluded that whilst behavioral inhibition and activation systems seem to work together to significantly predict mental disorders, lifestyle cannot.

## Introduction

Coronaviruses are a major group of viruses known to be responsible for a wide spectrum of diseases in multiple mammalian species, with subsequent effects of their viral infection leading to respiratory diseases such as the common cold, pneumonia, bronchitis, severe acute respiratory syndrome, and Middle East respiratory syndrome [1]. At present, the newly identified SARS-CoV-2 has caused a significant number of deaths worldwide, posing a serious threat to global public health [2]. Current approaches to prevent transmission of the novel coronavirus have included social distancing and quarantining. These necessary methods of isolation have subsequently resulted in restrictions of mobility, social interactions, and daily activities of the general public [3]. Restrictions of these kinds have been suggested to lead to challenges in psychological health, as well as of course general subsequent challenges in the navigation of social aspects [4]. So, although social distancing is a crucial intervention to the slowing down of destructive effects of SARS-CoV-2 social distancing has also had significant negative consequences of its own, one of which can be concluded to be the possible increase of mental illness, particularly given that the two factors noted to be increasingly important to mental illness and health (behavioral systems and a person’s lifestyle) are also significantly affected by the consequences of the pandemic [5].

Swinkels’s theory (2005) explains how brain-related personality traits are associated with the formation of life quality pathological disorders [6]. In fact, this theory investigated a wide range of mental pathologies in recent decades, which is a useful framework for understanding motivational and personality disorders in a variety of mental disorders, including depression, anxiety, and stress. Reinforcement Sensitivity Theory provides a neuropsychological explanation of personality, which is the main cause of many of the individual differences observed in personality. It is believed that the dominance and activity of each of these systems in individual leads to emotional status, coping styles and different reactions in individuals [6], which can greatly affect people’s lifestyles. In the meantime, adolescents are most affected due to their special physical and mental characteristics [7].

On the other hand, behavioral inhibition system processes information related to threat, and triggers anxiety. It inhibits behavioral progress, increases provocations, and directs attention in response to threatening (danger) symptoms toward the threat [8]. Athlete adolescents with a more active behavioral inhibition system are more vulnerable to stressful experiences and situations, as the sensitivity of their inhibition system increases. This system motivates the movement towards goals that lead to positive consequences, and impulse is the most important dimension in this system [9]. Behavioral activation system contributes to positive motivational tasks and avoidance behaviors in adolescent athletes. This system engages the individual in orientational behaviors and encourages one to take action that leads to a reward without even realizing the possibility of its negative consequences [10]. Therefore, given these cases, when adolescent athletes are restricted in home quarantine, they will experience a different lifestyle that can be harmful for them [11]. During the COVID-19 pandemic, adolescent athletes described an increase in depressive symptoms, decreased physical activity and quality of life compared to their peers in previous years [12].

In addition to the above-noted challenges (i.e., pubertal development, focus on exercise, food, and body image, sport-specific pressures, gender-specific pressures) outside of COVID-19 [13], the pandemic has created additional concerns for adolescent athletes. Indeed, the interruption of organized sports during the lockdown has been challenging with altered routines, uncertainty regarding training and competitions, and for most elite, their professional sporting careers [14]. Many of the psychosocial and physical benefits of sports must be weighed against the environmental considerations and minimizing the risk to athletes of acquiring COVID-19.

Furthermore, recent research on the psychological impact of the COVID-19 pandemic specifically on elite athletes suggests that levels of perceived stress and negative emotions during the lockdown (in 2021) are higher in athletes who experience changes in their: (1) motivation to compete (decrease from pre-pandemic); (2) stress-management (lowered coping abilities from pre-pandemic); and (3) post-pandemic performance expectations (maintain their usual high pre-pandemic standards, without allowance for COVID-19 interruptions) [15]. Conversely, positive emotions during the lockdown are higher in athletes who experience no changes in their competition motivation, stress-management, or post-pandemic performance expectations [15].

To investigate the effects of Covid-19 pandemic on mental health of adolescent athletes and so its proper management, it was necessary to measure its daily effects and consequences changes on adolescent athletes [16–18].

This information could be a great help to coaches, clinicians and families in using the right principles for improving the mental and physical health of adolescent athletes during Covid-19 pandemic. Although competitive sports always increase the level of physical activity in young ones, this level of physical activity can never be achieved on a daily basis. Thus, an appropriate approach should be adopted to increase the level of physical activity at early ages for example during the childhood and adolescence. (Title: Lifestyle and resulting body composition in young athletes,2021) During the COVID-19 pandemic, adolescent athletes described an increase in depressive symptoms, decreased physical activity and quality of life compared to their peers in previous years.

Quality of life can be considered as the most important part of every individual’s life. Adolescence is a time when a person is exposed to a lot of mental stress and tensions and this can endanger his/her quality of life. On the other hand, adolescents who do exercise and consider physical activity as a part of their everyday lives are more affected by these conditions due to the restrictions imposed by quarantine. During the COVID-19 epidemic, an increase in depressive symptoms, decrease in physical activity and quality of life are reported among adolescent athletes compared to their peers in previous years [19].It has thus become increasingly clear in recent decades that lifestyle factors contribute significantly to the ever-growing burden of chronic illnesses and diseases [20]. Understanding lifestyle factors that can come to affect our health only works to improve and reduce the prevalence of preventable diseases [21]. Specifically, planned education on lifestyle modifications can coincide with the reduction of general anxiety levels in patients [22]. Questionnaire scores on lifestyle and mental disorders display inverse relationships of statistical significance [23]. The use of diet, exercise, and behavior modification may come to not only provide physical health benefits to the patients utilizing them, but also psychological health benefits overall, reducing severe patient depression and anxiety in general [24].

The behavioral activation system (BAS) and the behavioral inhibition system (BIS) are two brain behavioral systems that also have a significant influence on these depressive symptoms [25]. The behavioral activation system is an arousal system that works by initiating behavior in response to conditioned stimuli for reward and punishment avoidance [26]. The neuroanatomical basis of the BAS has been indicated to be in the right dorsolateral prefrontal cortex, the right temporal polar region, the basal ganglia, the amygdala and the hippocampus (Fowles, 1980). This has then been supported by findings of greater left frontal brain activity showing higher BAS scores in investigated participants [27].

Results investigating BIS related anxiety in recent years have been found to be especially interesting. One study found a moderating relationship between disgust propensity and anxiety sensitivity in regards to physical concerns surrounding the fear of contracting COVID-19, lending a unique support for individual variation in the activation of the BIS [28]. Taken separately in fact, both this anxiety sensitivity and disgust propensity predict the fear of contracting COVID-19 [28]. These findings are important in that they create a reminder of the necessity of developing alleviation methods for anxiety in individuals fearful about contracting COVID-19, with specific consideration on these disgust reactions that follow [28], as because of anxiety and these disgust reactions, people come to adopt various unwanted lifestyle and dietary modifications under the influence of rumors (Roy, Tripathy, Kumar Kar, Sharma, Kumar Verma, & Kaushal, 2020) that might be of higher detriment. Thus, general psychological conditions of the public are significantly affected during the COVID-19 outbreak [29].

However, amidst the COVID-19 outbreak, little attention has been paid to the psychological and behavioral impacts this disease has had [30]. Current evidence indicates a high prevalence of mental health problems among patients, caregivers and healthcare providers with a recent study specifically showing anxiety and depression symptoms being at an all-time high among healthcare professionals working in the operation room [31]. Although there has been substantial attention to creating measures that identify the mental health care issues induced by the pandemic, understanding of the needs of the victims of these mental impacts has nevertheless been neglected [32]. Recognizing these challenges allows for strengthened mental health services during times of quarantine and isolation [3]. With public levels of anxiety-related symptoms increasing when a major infectious disease spreads [29]and heightened anxiety due to COVID-19 being found to linger in people for up to two weeks following any incidental contact with those believed to be possible hosts for the virus [28] the importance of promoting public awareness and scientific health education without the creation of anxiety and fear in the community is at an all-time high, especially since current methods might work to instead increase the worries and the demand on health services without any real indication [33].

Individual psychosocial factors should thus be thoroughly evaluated to identify risk and protective factors among Adolescent Athletes in order to guide the development and adoption of effective personalized mental health measures [34]. There are thus calls for increased awareness of the at-risk population as necessary targets for specialized psychiatric care and prompting continuous psychiatric intervention of this population during these outbreaks of life-threatening, epidemic-potential infectious diseases [35]. Epidemiological and clinical studies clearly delineate extensive reduction in the quality of life of persons with anxiety and anxiety related disorders [36] and previous studies have shown that lifestyle interventions can be utilized to increase antidepressant efficacy [37] but nothing has come to suggest the possibility of a mediating role of behavioural systems between the two, especially in the context of lifestyles Adolescent Athletes factors during the pandemic. The following research thus aims to investigate the relationship between behavioral systems, lifestyle and mental disorders in adolescent athletes during Covid19 pandemic, with the hypothesis that a negative correlation will be found between BAS and mental disorders. A positive correlation will be found between BIS and mental disorders, and finally a negative correlation will also be found between general lifestyle and mental disorders in adolescent athletes during Covid19 pandemic.

## Method

 Participants of the study included 130 Iranian male adolescent athletes residing in the Mazandaran province (one of the most affected areas of Iran during COVID-19), who were recruited through the three University study sites nearby (Farhangian University, Tarbiat Modares University, and Islamic Azad University).

Study inclusion criteria were: (1) aged 12 to 19 years old (“adolescence,” as defined by Rice, F.P., in Human Development: A Life-Span Approach) [38]; (2) completion of a physical illness history measure, assessed pre-study through participant self-report via the WHO Global Physical Activity Questionnaire (WHO-GPAQ) [39] (no disabilities or physical problems were reported by the research participants); (3) completion of a mental illness history measure, assessed pre-study through participant self-report via the WHO-Composite International Diagnostic Interview (WHO-CIDI) [40] (no specific, diagnosis of a mental disorder (e.g., trauma or depression), “Students Athletes” with Intellectual disability and “Students Athletes” with other nationality were reported by the research participants);; (4) sports history for at least 3 years, assessed via the WHO-GPAQ; and (5) written consent by the adolescents and their parents for participation in the research. The study exclusion criterion was: 1) lack of regular physical activity, assessed via the WHO-GPAQ. This criterion was included in order to differentiate between a lack of regular physical activity due to normal patterns (e.g., sedentary lifestyle), vs. a lack of regular physical activity due to changes during the COVID-19 pandemic assessment period (e.g., reduced exercise levels). Of note, all included participants had continued exercise during the pandemic (see details below). The amount of regular physical activity was tracked by both the adolescent athletes and their parents, and the accuracy of the level of activity was confirmed by the study researchers. Thus, all participant responses were equal in terms of the associations between activity and scores.

Of the eligible participants who were available during a COVID-19 quarantine period from June through August 2020 (N = 180), the Krejcie and Morgan Sampling Method was used to simplify the process of determining the sample size for a finite population [41], resulting in a calculation of *N* = 130 sample participants. Throughout the data collection quarantine time, all athletes practiced and played at home, or in socially isolated environments, depending on the training that the coaches designed for them. Thus, the athletes had continued exercise during the lockdown, albeit with modified routines and formats.

### Instruments

#### Behavioral Inhibition/Activation Systems Scale (BIS/BAS)

This questionnaire is a self-report questionnaire developed and validated by Carver and White (1994) [42]. It contains 20 items and two main scales: the scale of the behavioral inhibition system and the behavioral activation system. The BIS/BAS scales were associated with prefrontal cortical activity, affect, personality traits, and performance on reaction-time and learning tasks [42, 43]. The BIS scale includes 7 items related to anticipation of punishment, whilst the BAS scale has 12 items and three subscales: Drive (D), indicative of persistence in obtaining desired goals, Fun-Seeking (FS), indicative of willingness to seek out and spontaneously approach potentially rewarding experiences, and Reward Responsiveness (RR), indicative of anticipation and positive response towards rewards. Carver and White (1994) reported the internal stability of behavioral inhibition as 0.72 and its differential validity as 0.55 with anxiety. Cronbach’s alpha coefficient of inhibition scales and behavioral activator subscales were reported to be 0.78 and 0.81, respectively. Also, in the research of [44] Cronbach’s alpha coefficient of inhibition and behavioral activation systems was 0.88 and 0.87, respectively.

#### Miller and smith ‘s life style questionnaire

The following questionnaire contains 20 items in which participants answer each item using a five-point Likert-type scale (1 = strongly disagree, 5 = strongly agree). Validity and reliability of the questionnaire was measured (Cronbach’s alpha: 0.86) [45]Miller-Smith Lifestyle Assessment Inventory (LSI) consists of 20 items with a 5-point Likert-type scale that asks respondents how often the related items are applies to them, e.g. “I eat at least one hot balanced meal a day” and “I give and receive affection regularly”. Response choices range from (always) to 5 (never). Total scores range from 20 to 100 (Miller & Smith, 1988). Miller and Smith (1988) reported the reliability as α = 0.85. The AMOS (version 22.0, Chicago: IBM SPSS) was applied to analyze research data by SEM (P < 0.001) [46]. Cronbach’s alpha coefficient of Miller-Smith Lifestyle systems was 0.88 and 0.87, respectively [47].

#### General Health Questionnaire (GHQ-12)

The 12-Item General Health Questionnaire (GHQ-12) (Goldberg & Williams, 1988) consists of 12 items, each one assessing the severity of a mental problem over the past few weeks using a 4-point Likert-type scale (from 0 to 3) [48]. The Questionnaire includes 12 questions assessing symptoms related to psychological distress and general functioning, e.g. ability to face problems and make decisions. The score was used to generate a total score ranging from 0 to 36. The positive items were corrected from 0 (always) to 3 (never) and the negative ones from 3 (always) to 0 (never). High scores indicate worse health. Relationship between GHQ-12 with Beck Anxiety Inventory was 0.69 (Gao et al., 2004). In research of Qin, Vlachantoni, Evandrou, & Falkingham (2018) and Elovanio et al. (2020) the reliability of the GHQ-12 Cronbach’s alpha was 0.90 and 0.92 [49]. In order to determine the sensitivity, specificity and best cutting point, the Receiver Operating Characteristic Curve (ROC Curve) was used, and as a result, the best cutting point for the 12-item General Health Questionnaire (GHQ-12) was 3.5 with a sensitivity of 87. Feature percentage was calculated to be 60% [50].

Data was analyzed randomly selected using Pearson correlation coefficient and linear regression analysis with Enter regression model. Pearson correlation or simple linear regression analysis can determine if two numeric variables are significantly linearly related. A correlation analysis provides information on the strength and direction of the linear relationship between two variables, while a simple linear regression analysis estimates parameters in a linear equation that can be used to predict values of one variable based on the other [51]. the Pearson correlation coefficient, r, can take on values between − 1 and 1. The further away r is from zero, the stronger the linear relationship between the two variables. The sign of r corresponds to the direction of the relationship. If r is positive, then as one variable increases, the other tends to increase. If r is negative, then as one variable increases, the other tends to decrease. A perfect linear relationship (r=-1 or r = 1) means that one of the variables can be perfectly explained by a linear function of the other [52]. All data were entered into the Statistical Package for Social Science (SPSS) 24 software for analyses [53].

## Results

The sociodemographic variables of the Adolescent Athletes (*n* = 130) are presented in Table 1. In summary, athletes had significantly mean values of age, height, weight, and Body Mass Index (BMI; as calculated by the Centers for Disease Control and Prevention (CDC) BMI Percentile Calculator for Child and Teen [54]) (p = 0.001). However, the range of distribution for each of these variables is a tight cluster of scores, as detailed below, Adolescent Athletes had a significantly higher mean age of 15.7 years (Standard Deviation, *SD* = 0.8 years; range), mean height of 161.3 cm (*SD* = 7.6 cm), mean weight of 65.3 kg (*SD* = 1.9 kg), and mean Body Mass Index (BMI) of 21.2 (*SD* = 2.4, as calculated by the Centers for Disease Control and Prevention (CDC) BMI Percentile Calculator for Child and Teen (*p* = 0.001). The results of the normality test show that among the participants, the descriptive statistics follow the natural distribution. Because in all these stages, the significance value of the Shapiro-Wilk test [55]is greater than 0.05.

**Table 1:** Sociodemographic variables of the adolescent athletes

Variable	Athletes (*n* = 130)			
Age	**Mean**	**SD**	**T**	***p*-value**
	15.7	0.8	3.4	0.001
Height (cm)	161.3	7.6	2.4	0.001
Weight (kg)	65.3	1.9	2.6	0.001
BMI	21.2	2.4	2.5	0.001

*SD*  Standard Deviation, *cm* centimeters, *kg* kilograms, *BMI* Body Mass Index, as calculated by the Center for Disease Control and Prevention (CDC) BMI Percentile Calculator for Child and Teen

The Table 2 shows that there is a positive correlation of statistical significance between behavioral inhibition systems and mental disorders, and a negative correlation of statistical significance between the scaling components of behavioral activation systems, lifestyle and mental disorders in Adolescent Athletes at the 0.01 and 0.05 level respectively (Table 1).

**Table 2:** Mean, Std. deviation and matrix of pearson correlation between behavioral systems and lifestyle with mental disorders of the adolescent athletes during Covid19 pandemic

	Mean	Std. Deviation	1	2	3	4	5	6	7
**BIS**	16.63	2.451	1	− 0.18^a^	− 0.16^a^	− 0.19^a^	− 0.19^a^	− 0.24^a, b^	0.37^a, b^
**Drive**	6.96	0.866	− 0.18^a^	1	0.79^a, b^	0.88^a, b^	0.94^a, b^	0.39^a, b^	− 0.29^a, b^
**Fun-seeking**	15.51	0.961	− 0.16^a^	0.79^a, b^	1	0.78^a, b^	0.92^a, b^	0.39^a, b^	− 0.25^a, b^
**Reward responsiveness**	13.70	0.890	− 0.19^a^	0.88^a, b^	0.78^a, b^	1	0.94^a, b^	0.44^a, b^	− 0.33^a, b^
**BAS**	36.16	2.548	− 0.19^a^	0.94^a, b^	0.92^a, b^	0.94^a, b^	1	0.44^a, b^	− 0.31^a, b^
**Life style**	71.89	8.574	− 0.24^a, b^	0.39^a, b^	0.39^a, b^	0.44^a, b^	0.44^a, b^	1	− 0.22^a, b^
**Mental disorders**	18.79	1.378	0.37^a, b^	− 0.29^a, b^	− 0.25^a, b^	− 0.33^a, b^	− 0.31^a, b^	− 0.22^a, b^	1

^a^Correlation is significant at the 0.05 level. ^a, b^Correlation is significant at the 0.01 level. The table above shows that there is a positive correlation of statistical significance between behavioral inhibition systems and mental disorders, and a negative correlation of statistical significance between the scaling components of behavioral activation systems, lifestyle and mental disorders at the 0.01 and 0.05 level respectively

The Table 3 shows the predictability of behavioral systems and lifestyle on mental disorders. According to this table with Enter regression model, behavioral inhibition systems and activation systems can significantly predict mental disorders, but lifestyle cannot.

**Table 3:** Enter model regression analysis to predict behavioral systems and lifestyle (independent variables) on mental disorders (dependent variable) in adolescent athletes during Covid19 pandemic

Model	Standardized coefficients	t	Sig.	Collinearity statistics					
	**Beta**			**Tolerance**	**VIF**	**F**	**Sig.**	**R Square**	**Durbin-Watson**
**BIS**	0.31	3.84	0.000	0.93	1.07				
**BAS**	− 0.23	-2.6	0.010	0.79	1.25	10.61	0.000	0.202	1.86
**Life style**	− 0.048	− 0.53	0.590	0.78	1.28				

## Discussion

Amidst the COVID-19 outbreak, little attention has been paid to the psychological and behavioral impacts this novel virus has had on the public [30]. Current evidence however indicates a high prevalence of mental health problems among patients, caregivers and healthcare providers, with a recent study showing anxiety and depression symptoms being at an all-time high among healthcare professionals working in the operation room [31]. Recognizing the challenges faced by those plagued with anxiety and depression allows for strengthened mental health services during these times of quarantine and isolation [3]. With public levels of anxiety-related symptoms increasing when a major infectious disease spread [29]and heightened anxiety due to COVID-19 being found to linger in people for up to two weeks following incidental contact with those believed to be possible hosts for the virus [28], the importance of promoting public awareness and scientific health education without creating anxiety and fear in the community is at an all-time high. Unfortunately, however current methods suggest to instead work to increase worries of patients and demands on health services without any real indication of results [33]. predictability of behavioral systems and lifestyle on mental disorders. According to this table with Enter regression model, behavioral inhibition systems and activation systems can significantly predict mental disorders, but lifestyle cannot.

These results are consistent with research on the relationship between brain- behavioral systems and mental health [56, 57]. However, it should be noted that the values obtained in this study as the relationship between brain- behavioral systems and lifestyle, are lower than previous studies, but they were in the same direction. The reason for the lower intensity of the obtained relationship should not be interpreted as the reason for the weak relationship between these variables, as other studies have proven otherwise. Better explanations are probably related to the age and demographic characteristics of the subjects of this study. Whether “the relationship between brain-behavioral systems and lifestyle changes with the age of the subjects, the questionnaires used, the athletes’ BMI, and other factors” are important questions that need to be addressed in further researches.

The important point in this study is that any variable that can prove its relationship and role in the formation of lifestyle and subsequent reduction of brain-behavioral systems should be considered specifically. In justifying the relationship between brain-behavioral systems and lifestyle, similar research can be mentioned that is in line with the present one like [31]. As a result, they are more successful at coping with negative experiences than those with poor emotional control [58].

But it was not in line with research of Uhrich, Heggestad, & Shanock, 2021,

They stated that emotional intelligence can describe situations better and causes more positive emotions during occurrence of unpleasant conditions that disturb adolescents ‘feeling. Inhibitory behaviors cause a negative feeling in adolescents that makes them unable to control their emotions and reactions. On the other hand, individual’s motivations may reduce inhibitory behaviors due to the increase in the efficiency of emotional intelligence [59].

Suwalska et al., 2021also, in her research, pointed out that the use of brain behavioral systems such as social support, thought control and mental visualization leads to alleviation of stress and problems related to behavioral disorders [60]. also believed that when confronted with stressful sources, individuals employ the potentialities of their social and physical environment to control stress. The common point of these two beliefs is the emphasis on support as a result of using effective handling and consequently, higher mental health [61]. But other researchers in their research showed that support, if not logical and effective, causes tension in adolescent athletes and lack of desirable conclusions during competitions, they argued that the reason for this is that adolescents think that there is an eternal support for them, so they get into trouble after this support is dismissed [62]. In other hand researchers believe that in the case of facing mental stress, the faith and belief that those stressing factors can be controlled, reduce their effect, they emphasized on the role of evaluation in dealing with stressful situations and affecting individual health[63]. Also, Alloy et al., (2008) stated that high behavioral inhibition scores would predict the diagnosis of disorders such as anxiety and depression [64]. Fayazi & Hasani, (2017) investigated the relationship between brain-behavioral systems and anxiety, and their research showed that trait and state anxiety has a positive relationship with the behavioral inhibition system, but a negative relationship with the behavioral activation system [65]. High activity of the behavioral inhibition system is associated with high levels of anxiety, depression [66] and post-traumatic stress disorder, because the behavioral inhibition system increases provocation, attention, evoking emotional states of anxiety, passive avoidance, behavioral inhibition, silence, and negative emotions.

During the pandemic it has been reported that there is an increasing amount of patient complaints on psychiatric symptoms such as depression, insomnia and suicidal [67]. Social isolation during this pandemic has become detrimental to patients in that they can no longer see their relatives or companions anymore, with even families living in the same residence having to remain cautious to the possibility of catching COVID-19 from one another [68]. Data has also shown a significant relationship between compromised quality of life and the increasing prevalence of mental and anxiety disorders. Healthy lifestyle, including proper nutrition, exercise and physical training as well as activities in life however have, as expected, found to have an inverse relationship with the prevalence of mental health itself [23]. Given the suggested role of behavioral systems with mental health, the relationship between behavioral systems, lifestyle and mental disorders during Covid19 pandemic was investigated to better understand possible future methods of intervention as well as current states of reality. Our findings were consistent with previous literature published by [4, 5, 69, 70]. Our findings indicated a positive correlation of statistical significance between BIS and mental disorders and a negative correlation of statistical significance between BAS, lifestyle and mental disorders at the 0.01 and 0.05 levels respectively. Our analysis also indicated that whilst BIS and lifestyle factors can significantly predict mental disorders, BAS cannot.

### Limitations

The present study had the following limitations: The statistical population of the present study includes only adolescent athletes in the age group of 12–18 years and due to the focus on this age group, the results cannot be generalized to other age groups. In addition, the instruments used in the present study, in spite of being practical, may be limited in measuring variables. It is suggested that due to the different activities of brain-behavioral systems in different people, using the findings of the present study, the context of diagnosing psychological problems, that can affect the lifestyle of adolescent athletes, should be considered. Not only this helps identify individuals susceptible to this component, but also the personality qualities and biological systems associated with the disorder that help individuals and professionals identify these factors and make interventions based on their characteristics.

### Suggestions

It is suggested that factors such as anxiety, stress, depression and other psychological aspects which affect individuals’ lifestyle, given their behavioral systems, studies should be conducted on various age group like children, young people, middle-ages and elderlies, and then results of various age groups be compared.

## Conclusions

According to results of this study, the high score of the behavioral inhibition system in interaction with the lifestyle of adolescent athletes during Covid-19 pandemic period is likely to have a significant influence on behavioral problems of adolescent athletes. Performing training regarding psychological problems can play a significant role in reducing anxiety in dealing with the stressful life events of adolescent athletes during Covid-19. Encouraging adolescent athletes to participate in sports activities at home during quarantine and group counseling sessions are effective methods to promote mental and physical health and prevent mental health problems.

## Data Availability

The datasets generated and analyzed during the current study are not publicly available, as individual privacy could be compromised, but are available from the corresponding author on reasonable request.
